# Review of Miller’s Theory of Dental Caries

**Published:** 1885-01

**Authors:** 


### Review of Miller's Theory of Dental Caries.

BY THE AUTHOR.

I desire to give, in closing, a very short *resume* of the work which I have accomplished.

1. I convinced myself by the examination of some thousands of slides of carious dentine, that micro-organisms were always present, and that they, without any doubt, were the cause of various anatomical changes which were found to take place in the structure of the dentine during caries. (Here, of course, the question of priority does not suggest itself; Leber and Rottenstein, as is well known, were the first to give definite expression to this fact.)

2. I proved, at the same time, that the invasion of the micro-organisms was not, in the majority of cases, simultaneous with the softening of the dentine, but that large areas of softened dentine could be found that contained no fungi. Of all those who examined my preparations in America, no one, whatever his theory, ever once denied this fact. I concluded from this that the softening of the dentine went in advance of the invasion of the organisms.

3. I determined by analyses of masses of carious dentine, sufficiently large to give reliable results, that the softening of the dentine is of the nature of a true decalcification. That the decalcification of the outer layers is almost complete, and diminishes in degree as we advance toward the normal dentine. Furthermore, that the same relations maintain in dentine softened in a mixture of saliva and bread, or in weak organic acids; also, that in a mass of carious dentine the lime-salts had been removed to a much greater extent than the organic matter.

4. I maintained from the first that the softening of the dentine was produced by acids, for the most part generated in the mouth by fermentation. I had, however, no direct proof of this.

5. I *proved* that fungi exist in great numbers in the human saliva and in carious dentine, which have the power to produce acid under conditions which are constantly present in the human mouth. I determined this acid, for one of the fungi, at least, to be the ordinary ferment, lactic acid.

6. I produced caries artificially, which under the microscope cannot be distinguished from natural caries, by subjecting sound dentine to the action of these fungi in fermentable solutions.

7. I determined the influence of various antiseptics and filling materials upon the fungi of caries.

8. I isolated various forms of these fungi, and determined *in part* the conditions most favorable to their development, their characteristic reaction upon gelatine, their physiological action, their effect when inoculated into the system of lower animals, and their possible connection with certain obscure diseases generally attributed to the carelessness of the dentist.

My continual search has been after facts, and such facts as I have obtained I have presented before the profession, never putting before them either theory or speculation, nor anything which was not the result of severe and continued labor, and in this spirit I propose to prosecute this work, as well as any other that I may undertake in the interest of the profession.—*Independent Practitioner.*

NOTE.—Since writing the above, I have succeeded in producing death by septicæmia of both mice and rabbits, by injecting into the lungs saliva from the mouth of a perfectly healthy person.

